# The Validity and Reliability of the FANTASTIC Questionnaire for Nutritional and Lifestyle Studies in University Students

**DOI:** 10.3390/nu14163328

**Published:** 2022-08-14

**Authors:** María Teresa Murillo-Llorente, Renata Brito-Gallego, María Luisa Alcalá-Dávalos, María Ester Legidos-García, Javier Pérez-Murillo, Marcelino Perez-Bermejo

**Affiliations:** SONEV Research Group, School of Medicine and Health Sciences, Catholic University of Valencia, C/Quevedo nº 2, 46001 Valencia, Spain

**Keywords:** FANTASTIC questionnaire, university students, nutritional, lifestyle, validity, reliability

## Abstract

The FANTASTIC questionnaire is a scientific instrument that can be used by health professionals for quickly and effectively measuring the quality of life and lifestyle of people. It is a simple questionnaire that measures different dimensions including nutritional status, but the possibility of using it as a resource for studies in the nutritional field (regardless of its correlation with this) has never been considered, nor has it been used for studies in university populations. The aim was to validate the FANTASTIC questionnaire to report on the participant’s lifestyle in a Spanish university population by using a cross-sectional study. A sample of 501 participants was obtained. The study was approved by the Ethics Research Committee of Catholic University of Valencia, and written informed consent was obtained from all participants. Sociodemographic, lifestyle variables, habitual diet, and nutrition-related lifestyle were collected individually. Participants also completed the self-administered FANTASTIC questionnaire. The reliability analysis of the FANTASTIC questionnaire revealed a Cronbach’s Alpha statistic result of 0.797. The Kaiser–Meyer–Olkin (KMO) value was 0.786, with a significant Bartlett’s Test of Sphericity (*p* = 0.000). This shows that the FANTASTIC questionnaire has good internal consistency and good construct validity. A retest was performed in four weeks’ time, showing excellent intraclass correlation values. We consider the applicability of the FANTASTIC questionnaire for nutritional studies in Spanish university students to be appropriate, and most students have high scores in the nutritional aspects of the questionnaire, showing correct diet implementation and good cooking skills.

## 1. Introduction

The quality of life is a multidimensional and personal concept influenced, among other variables, by the environment, education, and the social group to which one belongs [1], which can be perceived as good or bad depending on the characteristics of the individual [2]. Quantifying it is not a simple task; thus, it is necessary to use instruments to measure quality of life in order to eliminate the possible bias of individual perception and to try to evaluate it as objectively as possible.

The FANTASTIC questionnaire is a scientific instrument that can be used by health professionals to quickly and effectively measure the quality of life and lifestyle of people by using a simple questionnaire without having to conduct exhaustive studies of the participant [3,4,5,6,7]. This questionnaire measures different dimensions, such as family environment, and friends; the participant’s activity, nutritional status, relationship with tobacco and toxic substances, and relationship with alcohol; aspects related to rest, sleep, and stress; personality type; and the internal vision of the person and their professional career [3,4].

The questionnaire was created in 1983 in Hamilton, ON, Canada, by Dr. Douglas Wilson (professor in the Department of Family Medicine at McMaster University in Hamilton) together with Ms. Ciliska (assistant professor in the School of Nursing at McMaster University) [8] when they felt that there was a need to create an instrument that could help health professionals ascertain people’s quality of life in a general manner in order to detect subsequent anomalies in the people they were assisting.

Originally, it was created as a mnemonic method for health professionals to grow closer to the patient and to know, in a simple and quick way, the family environment, professional career, and other important and relevant aspects of a person’s lifestyle. The questionnaire deals with a series of fields related to the person’s lifestyle habits that influence their state and lifestyle, as well as aspects such as their personality, which will influence their day-to-day behavior [8]. In each field, we can find different general questions for drawing further conclusions.

The score of the questionnaire ranges from 0 to 50. If the score is between 42 and 50 points, it is interpreted that the participant has their lifestyle under control, that it is excellent, and that it is being led correctly. If it is between 35 and 41, it indicates that he/she is close to the most appropriate healthy lifestyle, but even so, the lifestyle he/she leads is correct (but not excellent). When the result is between 30 and 34, they are leading a lifestyle right on the edge of what is acceptable as healthy. Between 20 and 29, it can be said that the lifestyle led by the participant is not acceptable and is below the limits that are accepted as healthy. Finally, if the value of the result is between 0 and 19, the lifestyle is not adequate. Table 1 shows the FANTASTIC questionnaire.

The evaluation of the results of the questionnaire depends to a large extent on the target population. Results obtained in a population with very different ages or circumstances may not be valid, and it is also of little help in assessing the quality of life of pregnant women since they experience a series of hormonal changes that can lead to weight gain and lifestyle changes during the nine months of pregnancy [9].

Up until now, the questionnaire has been used mainly for studies in adult populations and is always associated with chronic diseases such as hypertension, hypercholesterolemia, and diabetes mellitus II. However, the possibility of using it as a resource for studies in the nutritional field (regardless of its correlation with this) has never been considered, nor has it been used for studies in university populations. The aspects covered in the questionnaire are inadequate for analyzing risks in the case of patients or participants with chronic diseases or with a genetic predisposition to suffer from congenital diseases [10,11].

Although the questionnaire was created in order to evaluate people’s lifestyle in a quick and efficient manner, in 1983 (the same year in which the questionnaire was created), Dr. Douglas Wilson and his team broadly adapted it with more response options (a total of 5 with respect to the 3 in the original) in order to be able to use it in larger studies. For this reason, they called it the “Research Version” [12]. However, in most cases, it must be adapted for different population groups. The adaptations made have always been based on the original questionnaire and not on the previously named “Research Version”. In several countries, especially in South America, the questionnaire has been adapted for different populations analyzed, modifying the dimensions and questions asked of the patient or participant and subsequently adapting the quality-of-life classifications to the scores calculated with modified questionnaires [13,14,15,16,17,18,19].

One of the aspects studied by this questionnaire is the participant’s nutrition, in which it analyzes the balance of meals, breakfast, and the intake of certain nutrients such as sugar, salt, or saturated fats. It also analyzes the consumption of junk food and the patient’s self-perception of his or her weight. All questions related to nutrition are of great importance since, without going too deeply into the nutritional status, extremely important data will be obtained for the subsequent evaluation of their quality of life and are able to detect risks of suffering from diseases such as obesity or hypertension (related to question number 3 of excess sugars, animal fats, salt, and junk food) or malnutrition problems (related to questions numbers 1 and 2) [13,20,21]. In addition to the issues related to nutrition itself, there are background issues of alcohol, tobacco, and toxins that are also related to the person’s nutritional status [22,23,24,25,26,27].

Despite the fact that this questionnaire analyzes issues related to patient nutrition, no studies have been found in the literature in which the FANTASTIC questionnaire is used for nutritional analysis, and this is probably because this questionnaire analyzes all aspects as a whole to obtain an overall conclusion of the participant’s lifestyle, without making specific differentiations for each of the aspects. Therefore, considering the aforementioned need that requires adaptation for different population groups, we consider it necessary to conduct an in-depth study on the capacity of the FANTASTIC questionnaire to report on the participant’s lifestyle in a population range that has been rarely studied, namely the university population, with specific nutritional characteristics derived to a large extent from the lifestyle they lead during their studies. The university stage is considered critical, making it essential to carry out health-promotion interventions that promote a healthy lifestyle [28] because university students may acquire attitudes and behaviors from peers that could lead to unhealthy lifestyles [7].

## 2. Materials and Methods

This is a cross-sectional study producing data that were used to validate the questionnaire for the young Spanish population. Data collection used opportunistic sampling. The sample consisted of Spanish university students enrolled in any university in Valencia, Madrid, Barcelona, and Alicante, which are all cities of Spain. ERASMUS students with poor knowledge of the Spanish language that prevented them from correctly understanding the informed consent form and the questionnaire, pregnant women, and students with disabilities or metabolic disorders were excluded. To calculate the sample size, we used the usual expression for estimating a proportion with a 95% confidence interval and a maximum expected error of 10%, with a maximum degree of indetermination (expected proportion = 50%). A minimum size of 107 students was estimated, considering that the calculations have been adjusted for expected losses of up to 10% of the observations. In the end, a sample of 501 participants was obtained. From that sample, a pilot test was conducted with 86 medical students who voluntarily agreed to answer the questionnaire twice for the test–retest procedure, with a time interval of one month and also to test the translation and cultural adaptation.

The final sample consisted of students from nine universities, including both public (*n* = 5) and private (*n* = 4) universities. The students from Valencia and Alicante were invited to participate anonymously in the study at their respective campuses. They were informed of the purpose of the study, as well as all the data necessary for them to understand and sign the informed consent form. Once signed, the corresponding questions in this study were asked. On the other hand, for students from the universities of Madrid and Barcelona, a call explaining the study was made to contacts through acquaintances or relatives. In these cases, they were asked to sign the informed consent form and to send it by e-mail. After receiving the form, another telephone call was made to ask the study’s questions.

The study was approved by the Ethics Research Committee of Catholic University of Valencia (approval code UCV/2019-2020/161), and written informed consent was obtained from all participants. This study complies with the principles outlined in the Declaration of Helsinki [29]. Data were collected between October 2019 and May 2021.

### 2.1. Variables

To characterize the study’s population, sociodemographic data were collected, such as age, sex, the city where they studied, the area of knowledge of their studies, whether they worked and studied simultaneously, and their place of residence. Variables related to habitual diet and nutrition-related lifestyle were also collected based on an adaptation of a questionnaire that has been used in health surveys among university students in several countries [30], such as self-perceived cooking skills (0–10), the place of lunch or dinner (Home, Outside, and Alternative), whether they cooked themselves (Yes, No), the type of food they usually eat (Cooked from scratch, Prepared, and Prepared) and the type of usual diet (Normal, Low carbohydrate, Avoid meat and egg, Gluten free, Vegetarian, High protein, and Reduced meat). Participants also completed the self-administered FANTASTIC questionnaire.

### 2.2. Data Analysis

Data were entered and stored in an MS Excel file and then transferred to SPSS v.23 software (SPSS Inc., Chicago, IL, USA) for statistical analyses. The normality of the data distribution was determined using the Kolmogorov–Smirnov test. The data were presented using mean and standard deviation (SD). The data of the qualitative variables were expressed as absolute value of cases and as a percentage. For the analysis of continuous variables, the comparison between the values was made by unpaired Student’s *t*-test. The contrast between the categorical variables was performed using the normal Chi2 test or Fisher’s corrected Chi2 test, as appropriate, complemented with the analysis of standardized residuals in the case of statistically significant associations. Two-sided *p* < 0.05 was considered statistically significant.

For the validation procedure of the questionnaire in the analyzed population, an internal consistency analysis was performed using Cronbach’s Alpha statistic and a construct validity analysis, using the KMO test (Kaiser, Meyer, and Olkin), Bartlett’s test of sphericity, and a factor analysis using the extraction method of principal component analysis with a Quartimax rotation and Kaiser normalization. To assess the stability of the questionnaire, we use the test–retest method. Test–retest reliability was measured with the intraclass correlation coefficient (ICC) with a 95% confidence interval.

## 3. Results

After data collection, a total of 501 Spanish student volunteers were included in the study. The mean age was 20.5 years (SD = 3.4). A total of 182 (36.3%) were male and 319 (63.7%) were female. Table 2 describes the sociodemographic characteristics of the sample.

### Validation of the Questionnaire

The reliability analysis of the FANTASTIC questionnaire revealed a Cronbach’s Alpha result of 0.797, and it did not improve significantly when any of the questions were eliminated, indicating good internal consistency. Table 3 shows Cronbach’s alpha internal consistency values as well as the intraclass correlation coefficient for all domains.

The validity analysis showed a KMO (Kaiser, Meyer, and Olkin) test value of 0.786, and Bartlett’s test of sphericity indicated a significance of 0.000, indicating the feasibility of factor analyses. Factor analysis was performed using the principal component analysis extraction method with a Quartimax rotation with Kaiser normalization, which revealed the grouping of the dimensions into four large factors that explain 74.57% of the variance. The grouping of the items is shown in the rotated solution (Table 4) in which the order of the presentation of the variables was modified to facilitate the interpretation of the results.

Table 4 shows a significant grouping of the items in four factors: in factor 1, the items emotional and physical state, as well as tobacco consumption (closely related to both), are included. In factor 2, personality, family environment, and nutritional status are included. In factor 3, activity and studies are included, and in factor 4, alcohol consumption is included.

These results show that the FANTASTIC questionnaire has good internal reliability and good construct validity; thus, its applicability to Spanish university individuals is considered appropriate.

After an analysis of the responses to the FANTASTIC questionnaire from the sample of university students, a total of 174 (34.7%) had an excellent lifestyle, 260 (51.9%) were adequate, and 51 (10.2%) were acceptable. Only 16 students (3.2%) had a below-average lifestyle. No statistically significant differences were found by sex (*p* > 0.05; Chi Square Test).

Table 5 shows the results of the nutrition aspect of the questionnaire. Table 6 shows the results of the analysis of the variables related to lifestyle and diet of the sample. We did not find statistically significant differences by sex in any variable except for the type of food and the diet usually followed (Chi Square Test; *p* < 0.05). In the analysis of standardized residuals, it can be observed that, in the case of type of food, men mostly prefer to cook and women prefer to buy precooked or ready-made food. With respect to the type of diet, men tended to follow a diet for muscle mass increase and women tend to follow a diet with meat reduction.

## 4. Discussion

The university period is, for young people, a period of change characterized by greater autonomy and the acquisition of greater responsibility. This transformation leads to new interferences in the students’ environment, which in many occasions leads to less healthy behaviors such as the consumption of tobacco and alcoholic beverages and, in the most serious cases, to the consumption of illicit drugs [7,31,32]. In addition, the high demands and stress [33] caused by university studies can trigger the appearance of physical problems, such as the high prevalence of excess weight [34] and emotional problems [35,36,37,38]. Although students show a positive attitude and know what a healthy lifestyle is, they may not be able to implement it in their own lives [39]. Anderson and Good [40] state in their study that students with BMI within the normal range have better academic performance. Excess weight is associated with a sedentary lifestyle, fast food, and a lack of physical exercise. Sogari et al. [41] stated that students consume unhealthy food due to time constraints, high-calorie foods, and high prices of healthy products.

Many studies have used the FANTASTIC questionnaire to evaluate the quality of life of healthy people or even people with a certain pathology [14,16,35,36,37,42], but to date, no study has been found in the literature that used this questionnaire for nutritional studies. The FANTASTIC questionnaire applied to Spanish university students has shown, by performing an analysis of internal consistency, a Cronbach’s alpha reliability coefficient of 0.797, which indicates a good reliability of the test, a result similar to that of other studies [14,16,42] that found very similar internal consistencies despite the fact that the sample size was significantly smaller than ours in those studies.

The general evaluation obtained in the questionnaire applied to Spanish university students was to a large extent excellent or adequate. Only 16 students (3.2%) had a lower evaluation, and no significant differences were found between men and women. More than half of the students had an excellent lifestyle. These students generally claimed that university was not a problem that caused excessive stress for them, that they had a positive family and friendship environment, and that they usually had enough rest. It is striking that, in these students, their nutritional dimensions always had a high score, in contrast to what has been reported by other studies [43,44] in which they find that, with the university routine, adolescents begin to present practical and fast eating habits, with a clear preference for industrialized products; a low intake of fruits, vegetables, and legumes; and skipped meals.

In the case of the students’ personal self-assessment of their culinary skills, most of them rated themselves with an 8, claiming to know both the basics and more elaborate recipes. On the other hand, none of the students rated themselves below 5, claiming to know the basics and the essentials for survival. Those with the highest scores expressed satisfaction with cooking and sought to enrich themselves with new culinary knowledge, although they tended to repeat the dishes they cooked well. In this case, we also found no statistically significant differences between men and women. These results are clearly different from those found by Gema López et al. [44], who observed a deficit in culinary skills that could explain the deterioration of the eating pattern of the university students they analyzed.

Regarding mealtimes, it was observed that most students eat at their place of residence, citing economic reasons and a lack of time, as did the only other study that found and analyzed these issues in Spanish university students [44]. Students who eat out usually claim that these behaviors are a result of a lack of time for traveling. Students who cook usually live alone in a house or share it.

The most disconcerting result obtained is the type of food preferred by Spanish university students, since a difference was found between what was stated by men and what was stated by women. The possible combinations reveal that women prefer pre-prepared food or buying ready-made food, as opposed to men who prefer cooking to buying pre-prepared or ready-made food, in contrast to the preconceived idea about women and housework [45]. Currently, at college ages, our results seem to indicate that men are more inclined to cook than women.

Regarding the type of diet followed by students, the vast majority follow a normal diet and, in a much smaller proportion, other types of diets such as gluten-free or vegetarian diets. A minority group follows a muscle-mass-gaining diet (high protein) or diets with low carbohydrates, reducing meat or avoiding meat and eggs. Although we found statistical significance in dietary preferences between men and women, we were able to discuss these results as we have not found other studies that analyzed these or similar factors in the literature.

## 5. Conclusions

The overall rating obtained in the questionnaire applied to Spanish university students was mostly excellent or adequate. Most students eat at their place of residence, citing economic reasons and a lack of time, and students who eat out usually claim that this was due to a lack of time for traveling. Women reported a preference for pre-prepared food or for buying ready-made food in contrast to men, who preferred to cook; thus, currently, our results seem to indicate that men are more likely to cook than women at the university age.

The FANTASTIC questionnaire has a good internal reliability and construct validity; thus, we consider that it is applicable for nutritional studies in Spanish university students, most of whom have high scores in the nutritional aspects of the questionnaire and showed correct diet choices and good cooking skills. As this is the first time that this questionnaire has been applied for nutritional studies, our results suggest that this instrument has a good scoring capacity for this type of study, rendering it important for intervention programs for which its purpose is to promote lifestyles for improving health and the quality of life.

## Figures and Tables

**Table 1:** FANTASTIC questionnaire.

		2 Points	1 Point	No Point	Highest Mark	Your Mark
FAMILY AND FRIENDS	My communication with others is open, honest and clear	almost always	Sometimes	Almost never	2	
	I give and receive affection	almost always	Sometimes	Almost never	2	
	I get the emotional support that I need	almost always	Sometimes	Almost never	2	
ACTIVITY	Active exercise (30 min, e.g., Running, cycling, fast walk)	More than 4 times/week	2 times/week	Almost never	2	
	Relaxation and enjoyment of leisure time	almost always	Sometimes	Almost never	2	
NUTRITION	Balanced meals	almost always	Sometimes	Almost never	2	
	Breakfast daily	almost always	Sometimes	Almost never	2	
	Excess sugar, salt, animal fats, or junk food	Almost never	Sometimes	Almost daily	2	
	Ideal weight	± 4 kg	±8 kg	> ± 8 kg	2	
TOBACCO AND TOXINS	Tobacco use	None in the past 5 years	None in the past 6 months	More than 10 times/week	2	
	Abuse of drugs, prescribed and unprescribed	Almost never	Sometimes	Almost daily	2	
	Coffee, tea, cola	never	3–6 daily	>6 daily	2	
ALCOHOL	Average intake per week.	<2	2	>2	2	
	Alcohol and driving	never	Only occasional	Often	2	
SLEEP, SEABELTS AND STRESS	7–9 h sleep per night	almost always	Sometimes	Almost never	2	
	Frequency of seat belt use	always	almost always	Almost never	2	
	Major stressful events in past year	none	1–2	>3	2	
TYPE OF PERSONALITY	Sense of time urgency: impatience	Almost never	Sometimes	almost always	2	
	Competitive and aggressive	Almost never	Sometimes	almost always	2	
	Feeling of anger and hostility	Almost never	Sometimes	almost always	2	
INSIGHT	Positive thinker	almost always	Sometimes	Almost never	2	
	Anxiety, worry	Almost never	Sometimes	almost always	2	
	Depression	Almost never	Sometimes	almost always	2	
CAREER	Satisfied in job or role	almost always	Sometimes	Almost never	2	
	Good relationships with those around	almost always	Sometimes	Almost never	2	
				TOTAL	50	

**Table 2:** Sociodemographic characteristics of the sample.

	Mean ± SD or *n* (%)
Age (years)	20.5 ± 3.4
Male	20.3 ± 2.8
Female	20.5 ± 3.7
Sex	
Male	182 (36.3%)
Female	319 (63.7%)
Simultaneous work/study	
No	386 (77.0%)
Yes	115 (23.0%)
University (city)	
Valencia	329 (65.7%)
Alicante	79 (15.8%)
Barcelona	53 (10.6%)
Madrid	40 (7.9%)
Living in	
Home	350 (69.9%)
Apartment for rent	82 (16.4)
Hall of Residence	69 (13.8%)
Knowledge area	
Health Sciences	448 (89.4%)
Social Sciences	31 (6.2%)
Scientific/Technological	22 (4.4%)

**Table 3:** Reliability of the questionnaire.

	Intraclass Correlation Coefficient (*n* = 86)	Cronbach Alpha
Family and Friends	0.897	0.792
Activity	0.880	0.772
Nutritional Status	0.847	0.790
Tobacco and toxic substances	0.797	0.787
Alcohol	0.801	0.801
Sleep, rest, and stress	0.924	0.775
Type of personality	0.864	0.782
Internal Vision	0.822	0.794
Career/Studies	0.865	0.770

**Table 4:** Rotated matrix of components (own elaboration).

	1	2	3	4
Internal Vision	0.821			
Tobacco and toxic substances	0.565			
Sleep, rest, and stress	0.526			
Nutritional Status		0.721		
Family and Friends		0.665		
Type of personality		0.420		
Activity			0.766	
Career/Studies			0.682	
Alcohol				0.904

**Table 5:** Nutrition-related results of the FANTASTIC questionnaire.

	Global	Male	Female	*p*-Value *
Balanced meals	1.70 (0.5)	1.75 (0.47)	1.68 (0.55)	0.105
Daily breakfast	1.43 (0.8)	1.35 (0.79)	1.47 (0.74)	0.097
Intake of sugars, salt, saturated fats or junk food	1.23 (0.64)	1.19 (0.67)	1.25 (0.62)	0.373
Ideal weight	1.81 (0.49)	1.80 (0.45)	1.81 (0.51)	0.824

* Student’s *t*-test.

**Table 6:** Variables related to habitual diet and lifestyle related to nutrition.

		Male	Female	*p*-Value *
Skills Cooking	<5	16	19	0.507
	5	20	41	
	6	28	44	
	7	41	79	
	8	46	93	
	9	14	29	
	10	17	14	
Place of residence	Home	122	228	0.396
	Apartment for rent	30	52	
	Hall of Residence	30	39	
Simultaneous work/study	No	145	241	0.291
	Yes	37	78	
Place of eating	Home	131	211	0.286
	Outside	44	98	
	Alternate	7	10	
Self-catering	No	54	95	0.962
	Yes	128	224	
Type of food	Cooked from scratch	246	133	0.000
	Pre-prepared	24	52	
	Ready-made	12	34	
Diet	Normal	150	267	0.001
	Low carbohydrates	3	7	
	Avoiding meat and egg	3	3	
	Gluten-free	4	12	
	Vegetarian	5	7	
	High protein	16	5	
	Meat reduction	1	18	

* Chi Square test.

## Data Availability

Not applicable.
